# Book Reviews

**Published:** 1818-09

**Authors:** 


### CRITICAL ANALYSIS

### *OF RECENT PUBLICATIONS,*

IN THE

### DIFFERENT BRANCHES OF PHYSIC, SURGERY, &c.

*Observations on some important Points in the Practice of Military Surgery, and on the Arrangement and Police of Hospitals; illustrated by Cases and Dissections;* by JOHN HENNEN, Deputy-Inspector of Military Hospitals. Edin. 1818. 8vo. pp. 496.

*(Continued from p. 166.)*

*INJURIES of the blood-vessels* are next considered.

"How the vessels escape so often as they do, has been a source of great surprise among surgeons, and is truly wonderful. Balls buff along them, pass between them, and traverse their courses in all possible directions, but leave them unhurt. Their elasticity has been considered as in a great measure contributing to this effect, and no doubt it does. Something also may depend upon their being in a state of dilatation or otherwise when the missile passes across them, but still much is left unexplained in the attempt at accounting for these phenomena."

The author adverts to the propriety of tying large arteries when lacerated, although they may not bleed, and of securing the remote extremity as well as that next the source of circulation. Some important remarks follow on secondary hæmorrhage, and salutary cautions to young surgeons, who, from their adroitness in the dissecting-room, are led to suppose that there can be no difficulty in cutting down upon and securing the trunk of a wounded artery. From the fifth to the eleventh day, when the separation of the sloughs takes place, is another period of danger. The use of the ligature is next adverted to, "re-invented," Mr. Hennen observes, "by Paré, for it was known to Celsus."* The short cut ligature is spoken of in favourable terms; and the origin of it traced

* Celsus appears to have employed the ligature for stopping hæmorrhage in cases of wounds from accident; so did Galen, Paulus Ægineta, Avicenna, Guy de Chauliac, and many other modern surgeons, previous to the time of Paré; but they did not use it to secure the arteries after amputation: it is from his employing it for the latter purpose that praise is due to Ambrose Paré.

to Dr. Maxwell of Dumfries, who had adopted it in the year 1798, and recommended it to several other surgeons. The use of it was mentioned to Mr. Hennen in 1813, by Assistant Staff-surgeon Hume, as what he understood to be the mode of practice of an American surgeon. Gun-shot injuries often lay the foundation for aneurisms; a ball passing close to, or between, the artery and vein of a limb, but without primarily opening either, has so far predisposed the parts to sloughing, that an eventual varicose aneurism is formed. A case is related that appeared 61 days after the receipt of the injury.

"It came on, he said, in the act of running up stairs in the hospital, after having drank Lord Wellington's health. The extent of this patriotic draught I ascertained to be nearly one pint of brandy, and some quarts of strong Brussels beer, swallowed within three hours."

The means of cure proposed, were the abstraction of blood, cold lotions, digitalis, and rigid abstemiousness in diet.

"I heard little more of poor ——— except his dying groans. In an evil hour an operation of tying the external iliac had been proposed and performed some time after I had ceased visiting him. In less than 60 hours afterwards he was no more, gangrene having supervened immediately. Not a drop of blood had been transmitted to the limb, and the truth of my prognostic was publicly proved, by a communication which appeared between the vessels on dissection."

*Injuries of the nerves* are productive of another class of serious affections; as distressing pains, paralysis, oedema of the limbs, &c. An interesting case is related of a general officer who lost his arm by a cannon-shot:

"He was carried to an adjoining hovel, where the common amputation was performed under very unfavourable circumstances; the night was coming on, the supply of candles was scanty, and the enemy's shot were flying in all directions. The General was placed under my care on the day after the operation. The variety of cross accidents from fever and extensive sloughing, it is not within my purpose at present to enlarge upon; but the first attempt at clearing the ligatures, and making gentle pressure on them, was attended with pain so excruciating, as to leave no doubt that each included a nerve, or was in a certain degree connected with some large nervous filaments. This agonizing sensation was not felt except the ligatures were pulled at, and then not in the stump itself, but referred to the finger, thumb, wrist, elbow, or even to the external skin, of the lost arm, as one or other ligature might be handled. I have sometimes been led to think, that the General uniformly felt the same sensations when the same ligature was touched, as I generally made my attempts to extricate them in a regular succession, and his complaints were often of the same succession of parts. More attentive observation, however, convinced me that this was not the case; for, if any one was pulled with more steadiness than another, he complained of all the parts suffering pain simultaneously. One small ligature, if pulled in an oblique direction *inwards* towards the axilla, always gave him imaginary pain about the elbow or in the skin; but, if the same was pulled strongly and directly *downwards*, the fingers were complained of. He has, frequently after the smarting of dressing was over, with great accuracy pointed out on my arm the course of the internal cutaneous nerve, as the scite of his ideal pain; often he has described that of the external; and, on one occasion, I, with utter astonishment, had the general neurology of my arm and fingers traced by him. But, unless the ligatures were pulled at, he had no other uneasy sensations than those which usually occur in persons whose limbs have been amputated. Once only did I ever know him refer his pain to the seat of the sensorium itself. On that occasion, from using an artery forceps to the ligatures, on which the slide moved rather stiffly, I exerted a greater force than I had intended."

After various attempts at cutting, pulling, twisting, and a graduated and constant strain by means of appending small weights, or tying the separated threads of the ligatures over little quills of plaster, fully a year elapsed before the last ligature was removed.

The ensuing section contains many valuable remarks on some general affections of the system from wounds, and on the importance of a free ventilation of hospitals. This leads to the consideration of *Hospital Gangrene.* That this peculiar species of gangrene has existed ever since hospitals were instituted, will hardly admit of a doubt; but, unless we except the cursory remarks of La Motte, we have no distinct evidence of its having been noticed by surgeons as a specific disease, before the latter part of the last century, when an account of it appeared in 1783, in the posthumous works of Pouteau. Since then, medical men have been taught to form a more just estimate of the importance of the study of the nature of morbid poisons; this disease has consequently met with a due degree of attention, and its character has been more clearly ascertained. We at present merely give an abstract of some of the most important observations of Mr. Hennen, as we shall have occasion to reconsider this disease in a subsequent article, when the remarks we have to offer on the subject will be adduced.

Our readers, bearing in mind the experience Mr. Hennen must have had during the long and arduous contest in Spain and Portugal, and having, ere this, discerned his assiduity and talent for accurate observation, will peruse his account of this disease with a particular degree of interest. The principal source from which it is derived, was the hospital at Bilbao, after the battle of Vittoria. It was distant about four miles from the town, and six from the sea on the southern bank of a small but rapid stream, which runs through a fertile valley, gradually opening from the town of Bilbao to the Bay of Biscay. The soil in the immediate vicinity of the hospital was dry and gravelly, shelving gradually from the water, until, at the distance of a mile, a lofty ridge of mountains rose behind, and perfectly screened it from the winds. Good water was scarce in the immediate vicinity of the hospital. The prevailing winds, which were westerly and south-westerly, blew up the valley from the ocean, and a mild steady breeze usually prevailed. During the latter end of August and beginning of September, at which period hospital gangrene made its appearance, the thermometer ranged from 68° to 70°; but, towards the close of September, the mercury rose in the wards of the hospital to 74°. No epidemic disease occurred among the inhabitants during the period our army occupied this station. The building was in every respect well adapted for an hospital, but there was deficiency of many articles of comfort and convenience. The bedding was scanty; the wounded were obliged to lie on the straw upon the floor, much crowded together.

"In an hospital situated as I have described, (says Mr. Hennen,) I found, on my arrival from Vittoria, in the last week of August, 1000 wounded men; the larger number of whom had arrived in successive escorts from the same place, many in waggons and carts; but a large proportion was so slightly wounded as to be able to complete the journey (nineteen leagues) on foot."

"The sloughing sores had all been collected into a separate airy ward, on the second floor, and were reported to me as mild, and yielding easily to the treatment adopted; but, as I was well aware of the insidious nature of these cases in a large hospital, full to an overflow with gun-shot wounds, pouring in under all the circumstances of a siege, or a great battle, and of the confusion consequent on such events, I was prepared for fever of the worst kind, and of the most contagious nature."

"My fears were soon verified at the Cordeleria; for, in a few days, the whole hospital was overrun with gangrene, which I more particularly dated from the arrival of some fresh wounded men from Vittoria, of whom about thirty were in an advanced stage of the disease, which, it was said, first appeared upon the journey down. The ward appropriated to sloughing cases at once became a horrid scene; every sore in the house assumed a malignant character; and the deaths increased in nearly a three-fold proportion."

"Let us suppose that our wounded have all been going on well for several days, when suddenly one of our most promising patients complains of severe pain in his head and eyes, a particular tightness about the forehead, want of sleep, and loss of appetite, and that these feelings are accompanied with quickness of pulse and other symptoms of fever; his wound, which had been healthy and granulating, at once becomes tumid, dry, and painful, losing its florid colour, and assuming a dry and glossy coat. This is a description of the first stage of our Bilbao hospital gangrene; and, if a brisk emetic was now exhibited, a surgeon, not aware of the disease that was about to form, would be astonished at the amelioration of the sore, and the unusual quantity of bile and of indigested matter evacuated by vomiting."

If the incipient stage of the disease were overlooked, the febrile symptoms soon became aggravated, the skin round the ulcer assumed a florid colour, which shortly became darker, and at last black, with a disposition to vesicate; these changes would take place within twenty-four hours, and, *whatever might have been the original shape of the wound, it soon assumed the circular form.* It also acquired hard, prominent, ragged edges, giving it a cup-like appearance, while the bottom of the cavity was lined with a flabby blackish slough; the parts were always destroyed in a series of concentric circles, the centre of which was the part originally affected.

"The gangrene still advancing, fresh sloughs were rapidly formed, the increasing cup-like cavity was filled up and overtopped by them, and the erysipelatous livor and vesication of the surrounding skin gained ground, while chains of inflamed lymphatics could be traced from the sores to the adjoining glands, there exciting inflammation and suppuration, which often furnished a new nidus for gangrene. The face of the sufferer assumed a ghastly anxious appearance, his eyes became haggard, and deeply tinged with bile, his tongue loaded with a brown or blackish fur, his appetite entirely failed him, and his pulse was considerably sunk in strength, and proportionally accelerated. In this stage, the weakness and irritability of the patient was such, that the slightest change of posture, or the most delicate examination of the sore, put him to torture, increased by his inability to steady the limb, which, if moved at all from the bed, was seized with tremors and spasmodic twitches."

They were affected with the greatest impatience of pain and depression of spirits. Wounds long healed, broke up, and fell into a state of foul suppuration; the skin, although perfectly sound, which had been touched with a sponge employed in washing the gangrenous sores, ulcerated, and soon became itself a slough. A soldier just landed from England, who was under the influence of mercury for a venereal complaint, died within forty-eight hours after his admission, from the gangrene having seized an ulcerated bubo. Some had extensive local disease without any affection of the general system. It was often attended with hæmorrhage; and, when it was checked, separation took place in detached specks, not in the waving line of common gangrene. The bones were variously affected; sometimes they became carious; at others, the phosphate of lime was absorbed, and the bone was converted into a mass somewhat resembling cartilage.

"In the treatment of the Bilbao hospital gangrene, (says Mr. Hennen,) although the ulceration might, to a superficial observer, seem to claim the first notice, yet it was to the constitutional treatment that we paid particular attention."

Emetics and purgatives were freely employed; and, "on the supervention of typhoid symptoms, which, during the months of August and September, very early made their appearance, the cure was conducted on the same principles as guided us in the treatment of pure typhus."

"I need scarcely say, that a remedy so strongly recommended as venesection had early occupied our attention; but, previous to the month of October, the obviously typhoid type of the disease made us extremely averse from employing it."

"A favourable case for venesection at length presented itself; the result was strikingly advantageous, and the practice became general; indeed, the very patients themselves implored the use of the lancet, and, from that period to March following, we used no other remedy, either as a cure or preventive."*

In the local treatment a great variety of methods were employed. In general, the sores were covered with a large fermenting poultice; and, if there were great tension and inflammation of the limb, cloths dipped in saturnine solutions were applied; great care was taken to prevent inoculation, by destroying the tow, &c. used in washing any of the ulcers. When the treatment was successful, small florid specks, about the sixth or seventh day, appeared to break through the black sloughs which then began to loosen.

"In some cases, however, the sloughs were amazingly tenacious, and required a strong solution of lunar caustic. (In those cases, the diluted Fowler's solution of arsenic, looked upon as a specific escharotic by some, will be found very serviceable in cleansing the sores; but, whatever is used, no violence should be employed.) A much more important object than the separation of the slough, was the removal of the patient to an airy and separate ward, as no disease was more apt to recur than this."

* Mr. Hennen states, that, for the application and enforcement of this mode of treatment, the greatest praise is due to Staff-surgeon Boggie.

In this species of gangrene, amputation was never employed before separation began to take place spontaneously. In speaking of the disease, as it occurred at the Gens d'Armerie Hospital at Brussels, Mr. Hennen says,

"The fever was constantly present with the sloughing. I have reason to suppose, that the sloughing in some cases preceded the fever; but, in all the others, as nearly as could be traced by attentive inspection of the sores, particularly some weeks after the establishment of the hospital, both appeared at the same time."

*Mortification*, as occurring under ordinary circumstances, is considered in another section, as far as regards the practice to be followed in cases where the removal of a limb becomes the object. Mr. Hennen considers Larrey's division of mortification into traumatic and spontaneous of great practical utility, and agrees with him "that, when mortification is the result of a mechanical cause, and puts the patient's life in danger, we need not wait until the disorder has ceased to spread. Naval and military surgeons concur in giving a favourable account of the results of this mode of practice; indeed, the facts adduced by Larrey, in the third volume of his Memoires, are testimonials in favour of it that cannot be controverted. Many of the greatest improvements in science have been generally neglected at the time they were first advanced, from existing prejudices; or from the style of writing or character of the authors not favouring the promulgation of them. The same use has been made of scientific works of this kind, that scholars and churchmen have done with those of Burton and of Gale. The propriety of the above mode of treatment was very judiciously argued for in a work published in 1807,* by Mr. Curtis, a naval surgeon, who had adopted it as early as the year 1782.

"*Tetanus* (says Mr. Hennen,) is the last and most fatal affection incident to wounded soldiers; my observations have tended more to show what I could *not* trust to, than what I could place the smallest reliance on, when the disease was once fully formed."

He has never effected a cure in a case of the acute symptomic tetanus, except in one instance, in which the disease was removed by mercurial inunction; but the patient several weeks after died of "mercurial marasmus."

"Sweating, which some have imagined critical, I have seen excessive during the whole course of the disease, and attended with a most pungent and peculiar smell."

* An Account of the Diseases of India, as they appeared in the English Fleet and in the Naval Hospital at Madras, &c. 8vo. Edin. 1807.

This observation may, perhaps, be worth connecting with the opinion of the nature of the disease that appears to be at present most generally adopted,—that it arises from some affection of the spinal marrow. In several cases of disease of this part that we have witnessed, the body, and the urine particularly, have exhaled a strong odour of ammonia, which has been detected in the latter, by chemical analysis, in considerable quantity. The observation of Mr. Hennen, which we have quoted, appears to have some conformity with this singular occurrence, and is worth attending to in the investigation of the cause of this disease. The author says he has seen no symptom, amongst the great number detailed by writers, invariably present, except obstinate costiveness. In almost every case, the patients had been exposed to a stream of air blowing upon them; this had sometimes been cold, and at others of a high temperature.

*Amputation* forms the next subject for consideration. The propriety of immediate removal of a limb, in cases where the attempt to save it would be hazardous, is again adverted to. The author then treats of the circumstances which lead to consecutive amputation, and the period at which it should be executed. As soon as a subsidence of fever is effectually announced by a free and healthy suppuration; by the abatement of local inflammation; by a restoration of the skin to its functions, demonstrated by returning coolness and elasticity, particularly on the affected limb, we should proceed to perform the operation on those patients in whom no hope of an ultimate recovery without it can be entertained. Removal to a separate ward, and, if possible, to another hospital, is considered one of the most certain means of establishing their recovery. In amputating at the shoulder-joint, Mr. Hennen objects to the employment of pressure previously to the moment at which the artery is divided; he has performed this operation seven times, and never witnessed the most remote approach to dangerous hæmorrhage. A curious instance is related of the sacrifice of prejudice to vanity: A strenuous protester against the necessity of previous compression of the artery, performed the operation with one hand, while he compressed the artery with the other. Mr. Hennen's mode of performing the operation at the shoulder-joint appears to be a modification of that of La Faye: among others, he mentions one practical direction, which, by those who have seen it executed by inexpert operators, will be considered of particular importance: after the integuments are divided, and the flap formed, by throwing the arm backward, the long head of the tendon of the biceps is exposed; by dividing this tendon, and running the scalpel forward along the groove, its back lying in it as in a director, we are at once conducted to the joint.

We cannot, with justice to the author, proceed in the consideration of this division of the work; every page is replete with practical remarks of the highest importance; and here, also, we must terminate our review. The remainder of the volume is occupied with the consideration of injuries of particular parts: but a desire to convey to our readers some idea of the information that may be acquired from the perusal of it, has already led us beyond our limits. We cannot, however, conclude without observing, that this work will evince the degree of perfection to which military surgery has advanced, and the benefits our art may thence derive. These will exist when the events that led to them can hardly be distinguished through the obscurity of ages; and for the aid Mr. Hennen has afforded to the means of mitigating human sufferings, he will experience what may fully compensate for a life of toil—

"Volitabit vivus per ora virum."

---

*Surgical Observations: being a Quarterly Report of Cases in Surgery; treated in the Middlesex Hospital, in the Cancer Establishment, and in private Practice. Embracing an Account of the Anatomical and Pathological Researches in the School of Windmill Street.* By CHARLES BELL. Vol. II. Longman and Co.

*(Concluded from our last.)*

*ON the Cure of White-Swelling of the Knee.*—Mr. Bell, in this report, makes some observations on the indifference of manner with which this disease is usually treated; the feeble debate, whether deep issues, blisters to the sides of the knee, or a poultice of bread and water, should be employed; shewing that there is no sanguine hope attached to either method. From observing that, when a spontaneous cure takes place after the disease has advanced so far as to destroy the fine texture of the surfaces, either inflammation produces adhesion and obstructs the motion, or the bones are anchylosed, or there is, at least, some mechanical obstruction offered to the motion of the joint by the form of the bones,—he was led to suppose that the artificial production of some one of these states would be attended with the same beneficial effects. Thus we frequently observe that, when the motion of the knee is prevented, or when the hip-joint is removed from the acetabulum, or the inflamed surfaces separated in any other way, the excitement ceases, and there is a subsidence of the inflammation. These cases, as Mr. Bell observes, particularly when the knee is the seat of the disease, although they terminate in what is usually considered a favourable manner, generally leave the limb in a contracted state, and the joint always liable to inflammation from slight causes of excitement: it is, indeed, such an incumbrance, that patients at last are frequently disposed to suffer amputation to be relieved from it. A consideration of these circumstances induced the author to use means to prevent the retraction of the leg, by splints applied to the back of the limb; and to produce adhesion between the surfaces of the joint, by a seton passed through its cavity. Mr. Bell has shewn that passing a seton through a joint which has been long diseased is not productive of the excitement which ensues from the introduction of extraneous bodies when the parts are in the natural state. He relates two cases in which this mode of treatment was adopted. The subject of the first was a man, aged 24. The disease had existed nine years, and he had come to London for the purpose of having the limb removed. Leeches and cold lotions were applied after the introduction of the seton: the inflammation induced was inconsiderable; and he left the hospital, in a few months, to walk to his home, at a considerable distance in the country. The other was a woman, aged 30, who also desired to have the leg removed: she was equally benefited by this mode of treatment.

*Of the Axis of the Pelvis.*—Mr. Bell endeavours to call the attention of the profession to this subject, as being important in the practice of surgery as well as midwifery. He shews that the axis of the pelvis should be considered in two distinct points of view; the one described by a line continued from the centre of the brim to the centre of the outlet, and equi-distant from all sides of the pelvis, and therefore necessarily semi-circular; the other, the centre or axis of revolution of the pelvis itself. The latter is important in many respects; for, as the pelvis changes its position with the posture of the body, so, according to the manner in which we place a patient, will it be necessary to vary the application of instruments. The former Mr. Bell calls *the line of axis through the pelvis;* the latter, *the axis of the revolving pelvis.* The first should be attended to in the practice of midwifery, in the operations of lithotomy, and puncturing the bladder; the other is likewise intimately connected with those operations, in directing the introduction of the sound or staff, and making the incision into the bladder.

*Second Report on Hernia.*—In this report, Mr. Bell treats on the state of the peritoneal sac in that disease. His object is to point out the resemblance and colour of the peritoneal sac to the intestine; the effect of effusion into the sac; and some peculiarities in the sac of the congenital hernia. When this covering is distended with fluid, it much resembles in appearance the intestine; and, on being punctured, a fluid, various in colour, escapes, when the intestine, often considerably less in size than this distended sac, is discovered. It is evident that, in this state of parts, an inexperienced surgeon may be deceived; thinking he has returned the intestine, when he has only pressed the fluid out of the sac into the cavity of the abdomen. It will also contribute to render inefficacious the operation of the taxis; as by this, the pressure, being conveyed through the medium of the fluid, must be made equally on all the circumference of the protruded intestine; instead of doing it with that scientific precision, first downward, then upward, by which the return of the intestine is to be effected. Mr. Bell, however, thinks that this collection of fluid sometimes preserves the gut from injury during rude attempts at reduction.

In the operation for strangulated congenital hernia, the author reprobates the direction given by Mr. Cooper, to return the herniary contents by mere dilatation of the sac with the finger, without cutting it through. He also observes, that Mr. Cooper is wrong in stating that the thickening of the neck of the sac is a rare occurrence; and considers that his recommendation is to divide the parts only to within an inch of the abdominal ring; when the finger is to be introduced, and if the stricture be felt at the ring, the dilatation is to be made by insinuating the knife between the sac and the ring: but, if the stricture be in the tunica vaginalis itself, at the orifice towards the abdomen, the knife must be introduced within it, and the strictured part cautiously divided. Mr. Bell says, there is not one of his pupils for whom he would not tremble, in seeing them attempt the operation after such a method. We should not expect to see it ever attempted in this way by any man who thinks for himself; and we have always considered it as one of the proposer's attempts at refinement, at the expense of safety and utility.

Mr. Bell next makes some remarks on the consequences which arise from the sharpness of the stricture in this species of hernia; one of which, deserving of serious attention, is, that it quickly causes ulceration of the gut at the strictured part, acting as a ligature. The above report is worthy of serious attention; it contains many useful practical remarks.

*Remarks on the Operation of Lithotomy.*—Mr. Bell first adverts to the variation in the manner in which surgeons still perform this important operation. On going into a cutler's shop, he observes, as many gorgets are shewn to us as there are men of note in the profession, and all of various shapes, and with ingenious contrivances to make the operation quite safe; evincing the unsatisfactory manner in which this operation is still performed, and authorising a presumption that want of success in the usual way of executing it has given rise to this anxiety respecting the instruments.

The long uninterrupted chain of successful cases, which has occurred in the practice of some men, is not, Mr. Bell observes, to be considered as evidence of the advantage of any particular mode of operating, without inquiring whether or not they have operated in the general run of cases, or selected their subjects, rejecting those in which circumstances were unfavourable to its success. He also very justly remarks, that "we operate for hernia, in the most desperate cases, without regard to our own reputation: why should there be a different rule in the operation of lithotomy? It is a subject of regret that the character of the operation should be injured in our hands, but a more imperious duty commands us to do all.

Mr. Bell then proceeds to the principal object of this paper, which is to recommend the use of the knife instead of the gorget. This subject has been so fully discussed, that it is quite unnecessary to mention the arguments of Mr. Bell in favour of a mode of operation which has been so eminently successful. The result of the practice of Cheselden (who certainly did not select his subjects) is well known, and it might have been expected would have decided the question in favour of this instrument. Mr. Bell describes, with much accuracy and minuteness, the various circumstances attending the operation; but the practical remarks in this description will not admit of abridgment: we may, however, adduce an account of its principal steps, which are not generally so well known as those taken in performing it with the gorget; and, being thus briefly laid before our readers, may induce them to reflect on the subject:—

1. The staff is struck upon the stone, and made to rest in contact with it.—2. The point of the knife is struck into the left side of the perineum, just under the arch of the pubes; it is carried downwards by the side of the anus, and past it.—3. The fore-finger of the left hand is put into the wound, and the rectum pressed down; and the muscles and the internal fascia cut across.—4. The edge of the knife is turned up, and the membranous part of the urethra and the face of the prostate gland exposed.—5. The point of the knife is pushed into the groove of the staff, and carried forward, and the prostate gland is opened by a lateral movement of the knife.—6. The edge of the knife is directed by the fore-finger, and enlarges the wound of the bladder; the knife is withdrawn, and the fore-finger is thrust deeper, so as to touch the stone.—7. The forceps are introduced, and touch the stone.

---

*Observations on Phagedæna Gangrænosa.* By H. Home Blackadder, Surgeon. 8vo. Edin. 1818.

In a preface, Mr. Blackadder makes some remarks on the importance of the subject of his work; and deprecates the prejudice in favour of the paramount and almost exclusive importance many surgeons of the present day are disposed to attach to the daring parts of operative surgery; but, while he is willing to allow that such subjects are in themselves not without interest, he is still inclined to believe that they are most fitly ranked with the monstrosities of nature; and that they are calculated to attract attention, rather from their singularity, than from any great or general advantages to be derived from them. The one shows what Nature can sometimes do of herself; the other, what injuries she can put up with from the hand of man; and, doubtless, both are objects of laudable curiosity. "But who," continues Mr. B. "could wish to see his art come to that pass, when only such credentials as contain a list of the number of carotid arteries, hip-joints, &c. he has operated upon, are considered valid for the reception of a surgeon into the confidence of the public?" This reminds us of a sentiment of Mr. Hunter: he said, he never approached a patient to perform an operation, without feeling humiliated; it was like the means a savage employs to accomplish his ends; what a man of a cultivated mind would execute with ease and gentleness, is by him done with force and violence. How peculiarly expressive is this, coming from such a man as Mr. Hunter. It speaks more than all the volumes that have been written on the advantages of science, and the beauty of modesty. It is itself a code of professional morals, and the τὸ καλὸν at which e all should aim.

The observations contained in this work, Mr. Blackadder states, were arranged as early as the year 1814, with the intention of having them inserted in a periodical publication; but a circumstance withdrew his attention from the subject, and, till a short time since, he wanted leisure or opportunity for again reverting to it.

"Phagedæna, or Phagedenic Ulcer," the author says, "is a term which has long been in use with writers on surgery, in treating of the history and cure of ulcers; and has been employed to designate those foul and irritable sores which exhibit a constant disposition to spread or enlarge; and which, in their progress, are frequently marked by an evident loss of substance. By modern writers, its application has been more restricted; being generally confined to those spreading ulcers which are not confined to any one texture, but which most commonly have their site in the integuments and cellular substance."

He proposes an arrangement of this kind of ulcer into the following species:—*Simplex*, *Cahectica*, *Venerea*, *Hydrargyrosa*, *Herpetica*, *Cancrosa*, and *Gangrænosa*. This arrangement is obviously imperfect, which the author admits, and it is also in other respects objectionable; but, as he purposes to treat on the last species only, we do not think it necessary for us to make any particular remarks on it.

This work is divided into two parts. The first, containing the history and cure of the disease, deduced from his own observation and experience; the second, an investigation into the history and treatment of it, as it is to be found in the writings of various ancient and modern authors.

"Phagedæna gangrænosa," says the author, "is the name by which I mean to designate that disease which, in this country, is usually denominated hospital gangrene, and which is familiarly known to the French surgeons under the appellation of "*la gangrène humide des hôpitaux*." It has also been taken notice of by different writers under various other names; such as the putrid, scorbutic, contagious, malignant, gangrenous ulcer, hospital sore, *pourriture d'hôpital*, &c."

He apologises for making use of a new term; but considers that, if the view he has taken of this affection be correct, it was no less requisite than allowable. That which he has adopted is strictly proper, as expressing the principal characteristics of the disease. After some general observations on the rapid progress, extensive ravages, and fatal tendency of this malady, Mr. Blackadder proceeds to give an account of Passages, a small sea-port town of Guipuscoa, in the province of Biscay, where he first became acquainted with it. Passages is situated on the side of an inlet from the sea, which is surrounded by high hills; and, from an entrance extremely narrow, it extends to the distance of about three-quarters of a mile, and terminates in a large natural basin, from which the rising grounds gradually recede. This basin, though now nearly choked up with mud, was once a principal rendezvous for the Spanish navy. The long narrow street, or lane, of which the town principally consists, had been originally cut out of the basis of a hill. In several places, it lies so low as to be on a level with the sea at high-water mark. The population had usually been from about sixteen to eighteen hundred; but, after the destruction of St. Sebastian, the number of inhabitants, including the British army, amounted to seven thousand. The town was so completely filled up, that the cellars, stables, and even the arch-ways, were, during the night, promiscuously occupied by men, boys, mules, and horses. The climate, during winter, is cold, moist, and changeable. During that in which the town was occupied by the British troops, it rained almost incessantly. The thermometer was seldom higher than 55°, and less frequently so low as 32°. Fuel was extremely scarce. From this town being in the immediate rear of our army, and affording the accommodation of a convenient harbour, it became the place at which all re-inforcements were landed, and to which such of the sick and wounded, as were likely to require a considerable time before they could be fit for active service, were sent.

Mr. Blackadder was, in November 1813, appointed resident medical officer of a division-hospital in this place. About this period, three patients were admitted with simple flesh-wounds of the lower extremities. They were young, healthy, stout-looking men, and were able to walk about without experiencing much inconvenience. He remarked, on removing the dressings from their wounds, that the nature of the discharge, and the general appearance of the sores, were somewhat different from any thing he had before witnessed. This proved to be the commencement of hospital gangrene. He says,

"Upon laying these cases before Mr. Baxter, (surgeon to the Forces,) I soon learned that I had to attend a disease with which I was practically, and in a great measure theoretically, ignorant. I had also the mortification to learn that it had made extensive ravages in other hospitals; that medical officers were much divided in opinion, both with regard to its nature and method of treatment."

He was instructed to use a lotion, composed of nitrate of potash dissolved in vinegar, which was to be kept constantly applied to the sores. This application, which was continued for three days, occasioned violent pain, while the disease was in no degree arrested in its progress; but, owing to the removal of these patients to another hospital, he was prevented any longer watching the progress of their complaint. About three weeks after these patients were sent away, a number of men were admitted, most of whom had been recently and severely wounded. It was discovered that four of them were affected with gangrenous phagedena; and, according to their own account, their wounds had been in that state from six to ten days previous to their admission into this hospital. From these cases, the disease was communicated to every ward in the hospital; and, in less than two weeks, a great proportion of the open sores had become affected with it.

Every known means, every thing that was likely to prove beneficial, was had recourse to. In some cases it was attacked through the medium of the general system, in others by topical applications; but with equal want of success. The state of the sufferers is described by Mr. Blackadder in a manner which evinces his sympathy with them, and the exertions he made for their relief; and the line of conduct that should be adopted by medical attendants in such circumstances is traced with much judgment, spirit, and decision. Seeing that all the measures hitherto employed hardly even mitigated the disease, he says, "It appeared that the only way of arriving at a more effectual method of treatment, would be still more carefully and minutely to watch the progress of the complaint through its different stages, and thereby endeavour to acquire a more intimate knowledge of its character." By these means he at length became satisfied of the truth of the following particulars:

"1. That the morbid action could almost always be detected in the wound, or sore, previous to the occurrence of any constitutional affection.

"2. That the constitution did not, in several instances, become affected, until some considerable time after the disease had manifested itself in the sore.

"3. That, when the disease was situated on the inferior extremities, the lymphatic vessels, and glands in the groin, were observed to be in a state of irritation, giving pain on pressure, and were sometimes enlarged before the constitution shewed evident marks of derangement.

"4. That the constitutional affection, though sometimes irregular, was in many cases contemporary with the second, or inflammatory, stage.

"5. That all parts of the body were equally liable to become affected with this disease.

"6. That, when a patient had more than one wound, or sore, it frequently happened that the disease was confined to one of the sores, while the other remained perfectly healthy; and that even when they were at no great distance from each other."

The first of the above series of observations differs from what has been related by some writers on this disease. Professor Brugmans, of Leyden, in a Memoir, which obtained the prize given by the Society of Sciences at Harlem in

1814,* says, "Before any change in the wound is perceptible, the patient complains, sometimes a few hours after entering the hospital, of a most disagreeable sensation throughout the whole body, but particularly the arms and legs; lowness of spirits, and disinclination for food, follow; the tongue is dry, and frequently covered with a yellowish coat; the thirst is inconsiderable when the appetite ceases; the pulse becomes unequal, small, and accelerated."—Moreau also absolutely asserts, that the constitutional symptoms precede the local; and so does Mr. John Bell, in his Principles of Surgery. It is difficult to discover, from the writings of the greater number of the French surgeons, what opinion they held respecting the order in which the symptoms occur. They, however, in general, agree in this—that topical management is of the first importance in checking the progress of the local disease.

Mr. Hennen, when treating on hospital gangrene, in his work on Military Surgery, says, "I have reason to suppose that the sloughing, in some cases, preceded the fever; but in all the others, as nearly as could be traced by attentive inspection of the sores, both appeared at the same time." (P. 253.) In another place he states, that "some had extensive local disease without any general affection. These cases I have principally observed to occur from the inoculated slough, among the attendants, who occasionally respired a purer air than the patients; and among the assistants, whose accidental scratches were best treated by destroying the part with nitrated silver."—"The slightest scratch of the dissecting knife festered; ulcers, whether simple or constitutional, became gangrenous; wounds, long healed, broke up; nay, the skin, although perfectly sound, which had been touched with a sponge employed in washing the gangrenous sores, ulcerated, and soon became itself a slough. This was observable among the orderlies and nurses."

Professor Brugmans states, that, in the year 1799, a quantity of lint was purchased in France, to be distributed in the various hospitals of Holland. Wherever it was used in dressing ulcers, a very violent hospital gangrene was the consequence. The fact was inquired into, and it was found that, in the place from which the lint had been brought, they were in the habit of washing the bandages, and other articles used in hospitals, in order to arrange them, and sell them as new lint. Pelletan states, that he saw the disease thus disseminated; and Pouteau, that it has been manifested frequently

* Inserted in the 34th volume of our Journal.

in patients by the application of surgical instruments which had touched an ulcer infected with it.

From the above-mentioned facts, and from that contained in the sixth of the series of observations of Mr. Blackadder, which is confirmed by the testimony of Professor Brugmans, Dr. Rollo, and Mr. Hennen, it is evident that gangrenous phagedena arises from a specific morbid poison, and that constitutional derangement is not necessarily connected with it; consequently, that the first-mentioned observation of Mr. Blackadder may be correct *in the greater number of instances.* Thus far we agree with the author; but we are not disposed to accede to that as being the *absolute* law of the progress of the disease, considering that the testimony of some accurate and judicious observers would lead us to form opposite conclusions. We postpone the further consideration of this question, until we arrive at that part of the work in which the author expressly treats on the history of the disease.

Mr. Blackadder submitted his opinion on this subject to a senior medical officer; but he was informed that gangrenous phagedena was generally considered a *constitutional disease.* On inquiry, he found that the mode of treatment, modified by the various theories which different individuals had adopted, was particularly directed to the diseased actions of the system.

"Notwithstanding," says the author, "the discouraging impression which this information was calculated to produce, it was not sufficient to remove the conviction which I felt in the accuracy of the preceding observations; and I could not avoid taking another opportunity of stating it as my opinion, that, if any thing could be found effectually to destroy the diseased action in the sore, it would also be found to effect the greater part of a cure; and that, were it consistent with the present ideas of medical officers in the army to treat this disease agreeably to the aphorisms of Hippocrates, 'that diseases which medicine will not cure must be referred to the knife, and, if that prove unsuccessful, *recourse must be had to cautery.*' I did not entertain a doubt, but that such a practice would be followed by the most happy events.—The employment of the cautery was, on this occasion, considered inexpedient; but I was given to understand that I was at liberty to use any remedy that was not inconsistent with the general practice and opinions of British military surgeons. Under these circumstances, the oxide of arsenic occurred to me as an article that was probably possessed of powers sufficiently active for effecting the purpose intended, and of supplying the place of a more powerful, though less gentle, remedy."

This, however, was not contained in the apothecary's stores; but he obtained some of Fowler's solution, which was employed diluted with an equal quantity of water. Two severe cases in the inflammatory stage of the disease were first selected. The patients were directed to keep their sores constantly moist with the solution, and to renew the lint at least once in two hours. They were supplied with opium, to take in case the pain produced by it should become excessive.

"On visiting my patients next morning," says Mr. B. "it was impossible not to be struck with the change in the expression of their countenances—from that of acute pain, mingled with despair, to that of ease and gratulation. Upon examining their sores, I found them completely dried up, and covered with a dark, semi-transparent, and insensible slough, of a somewhat horny consistence."

The farther progress of the disease was evidently and completely arrested, and, by suitable applications for assisting nature in throwing off the slough, and in cicatrizing the sores, they were, in no great length of time, completely cured, without having used any internal medicine, farther than what might be occasionally required to prevent constipation of the bowels. Mr. B. continues,

"From this period, I never saw an instance in which this method of cure failed of success, when the remedy was timely and properly employed. Patients, whose sores had resisted, as was said, almost every other treatment, were admitted from other hospitals, and cured by it; and it was also, as I was informed, ultimately introduced into other hospitals, and proved equally successful."

We now arrive at the second chapter, in which Mr. Blackadder enters more fully into the consideration of the history of gangrenous phagedena. The development and progress of the disease are described with much minuteness, and, we doubt not, with equal accuracy; and the character of it, as depicted by him, accords with the opinion he has formed of the nature of the affection. We would gladly transcribe the whole of this account, but our limits confine us to those points which tend to prove the origin of the disease from the local contact of a specific morbid poison. We must, however, state that these extracts will convey a very imperfect idea of the value of this part of the work; and of the accuracy, penetration, and disposition carefully to investigate facts in their series and order, so manifest in the character of the author. After observing, that the general appearance and *odour* of the sore is such as cannot be mistaken by a person accustomed to witness the disease, he says,

"When the morbific matter, which produces this disease, has been applied to some part of the surface of the body from which the cuticle has been removed, as by a blister, the first morbid appearance which presents itself is that of one or more small vesicles, which are filled with a fluid, in some instances having a watery appearance; while in others it resembles a bloody serum. The situation of the vesicle is generally at the edge of the sore: its size is not unfrequently that of a split garden-pea.

"During the formation of the vesicle, the patient is generally sensible of a change in the usual sensation of the sore, which he cannot well describe, but which is accompanied by an occasional painful sensation, resembling the stinging of a gnat."

The vesicle, in one or two days after its formation, assumes the appearance of a slough, which begins to extend, and spreads with more or less rapidity, until it occupies the whole surface of the original sore; and, when left to itself, there is little or no discharge, but the slough acquires daily a greater degree of thickness. When the formation of the slough has been interrupted, the stinging sensation becomes more acute, phagedenic ulceration quickly commences, and in a few hours a very considerable excavation will be formed. The edges of this cavity are well defined. The lymphatic vessels are, in many cases, irritated. The soft parts in the vicinity of the sore become painful, tumified, and indurated; and, in persons of plethoric and irritable habits, an attack of acute inflammation supervenes. This generally subsides within from two to five days: the slough on the sore becomes more moist; matter is discharged around its edges; it then begins to separate, and is soon thrown off, but only to prepare the way for an extension of the disease, by a continued process of ulceration, and a recurrence of the last-mentioned symptoms.

This account of the progress of gangrenous phagedena agrees exactly with that of the disease termed *sloughing phagedena* by Dr. Adams,* or the *nigrities serpens* of Celsus. There is an important symptom, described by Brugmans as characteristic of hospital gangrene, which is not mentioned by Mr. Blackadder: he observes, "there is often found, at some distance from the ulcer, *a circle of a deep red, which extends more and more in concentric lines.*"

The author then describes the appearance of the disease when it supervenes upon an old sore, where a considerable depth of new flesh has been formed: "it is then seldom detected until it has assumed the form of a small dark-coloured spot on some part of the surface, and generally at the edge

* Treatise on Morbid Poisons, ch. v. p. 57.

of the sore, as has been noticed in the foregoing variety. But, by careful observation, it has been ascertained in several instances, that it commenced, as in the former instance, in the form of a vesicle." The future progress of the disease is nearly the same in both cases.

"When the morbific matter has been inserted under the cuticle by puncture, superficial incision, or scratch, the progress of the disease, in its early stages, can be more accurately observed than in any other instance; but, as the symptoms which are exhibited during the first nine or ten days after vaccine inoculation has been performed are so well known, and have been so frequently described, all that seems necessary on the present occasion is to observe that, with the following exceptions, the two diseases bear so striking a resemblance to each other as to render a particular enumeration of the symptoms unnecessary. The primary inflammation in gangrenous phagedena commences at the end of the second, or early on the third, day; the inflammation is at its height about the sixth: when the scab begins to form in one disease, phagedenic ulceration commences in the other."

Here, however, we have the specific distance of the inflammation attending the action of a morbid poison evidently referred to.

"When it supervenes upon a recent gun-shot wound, the discharge is at first found to be lessened in quantity, and to have become of a sanious nature; the sore has a certain dry and rigid appearance; its edges are more defined, somewhat elevated and sharpened; the patient is sensible of a change in the usual sensation in the sore, and he also complains of the occasional stinging before mentioned. The integuments at the edge of the sore become inflamed, and the surface of it assumes a livid or purple colour, and appears as if covered with a fine pellicle: this gradually increases in thickness, forming what has been termed a slough. An offensive matter then begins to be discharged, and the edges of the sore, which are usually jagged or pectinated, become extremely irritable, of a deep red colour, and dotted on their inner surface with numerous, small, elevated, and angry-looking, points, which may be considered as one of the characteristic marks of the disease."

Some observations follow on the variation of the degree of local inflammation, from the habit of body of the patient, season of the year, &c. and the appearance the sore assumes in different parts; after which the author proceeds to the consideration of the constitutional symptoms, and which he states never to have had an opportunity of seeing precede the local,—"unless it be held, as an exception, when the disease has supervened upon a stump, after amputation has been performed on account of the previous effects of the disease."

2

"The period at which the constitution begins to exhibit symptoms of irritation is extremely irregular: sometimes it is so early as the third or fourth day, and sometimes even so late as the twentieth."

It assumes more of an inflammatory or typhoid character, as the particular causes producing these modifications have been predominant. These are described with much accuracy and judicious discrimination; after which, the author points out the diagnostic symptoms of the local disease, and observes that it is liable to be confounded with phagedena cahectica, the mercurial phagedena, the scorbutic ulcer, and that morbid action which supervenes upon a wound when the constitution becomes affected with some acute disease.

The origin of gangrenous phagedena is next considered. After mentioning the opinions generally adopted, Mr. Blackadder says,

"Whatever may be the source of this disease, it is, at least, sufficiently ascertained that, when it occurs, its progress is only to be prevented by the most rigid attention to cleanliness; and by insulating the person or persons affected, so as to prevent all direct intercourse between them and the other patients; for, so far as I have had an opportunity of observing, ninety-nine cases in the hundred were evidently produced by a direct application of the morbific matter to the wounds or sores, through the medium of sponges, tow, water, instruments, dressings, &c.; while others, who were, in every other respect, equally exposed to its operation, never caught the disease. In attempting to prove this by experiment, I have placed three patients with clean wounds, (one of which was an extensive wound of the thigh, another a wound of the leg, and a third a stump of a thigh,) alternately between three other patients severely affected with the disease. They lay in a part of a ward which was appropriated for patients who were labouring under the disease, and of whom there were, at the same time, a considerable number. Their beds were on the floor, and not more than two feet distant from each other; but all direct intercourse was forbidden, and they were made fully aware of the consequences that would follow from inattention to their instructions. The result of this trial was, that not one of the clean wounds assumed the morbid action peculiar to the disease, nor was the curative process in any degree impeded. I have likewise had an opportunity of witnessing a similar result in two instances of this disease occurring after amputation; in both of which cases the patients lay in small apartments, each containing from six to eight patients with healthy wounds; but in neither case was the disease disseminated, although it proved fatal, in both instances, to the individuals affected with it."

We have before remarked, that the testimony of surgeons who have had opportunities of investigating this subject, and of the accuracy of whose observations we are disinclined to doubt, is in opposition to that of Mr. Blackadder. Although disinclined to multiply authorities unnecessarily, we cannot forbear adducing the following circumstance, related by Professor Brugmans.—"At Leyden, at the end of the summer of 1798, in the French military hospital, the hospital gangrene prevailed in one of the lower wards, while the other patients, attacked with slight wounds, and placed above this room, in an airy attic, were free from the evil. The surgeon on duty thought it expedient to have a hole cut in the floor, in order to procure a vent for the foul air, in its progress through the roof. Thirty hours afterwards, the three wounded men, who lay in the garret nearest the aperture, were attacked with gangrene, which soon spread throughout the whole room."

Much benefit will accrue to practice from the view Mr. Blackadder has taken of gangrenous phagedena; and in those cases particularly in which it is primarily a local affection, (and that it may be so in many instances we perfectly concur with him,) the advantages that will be derived from his mode of treatment, above that of any previously adopted, (if we except the actual cautery, employed by the French, which M. Roux calls an *heroic r medy*, and the want of permission to use which in the English army is so feelingly regretted by Mr. Blackadder,) are evident, and of the utmost importance; but, in the present state of our knowledge respecting it, we consider that the arrangement of a military hospital, on the principle that it cannot be disseminated through the medium of the atmosphere, and in the manner in which those diseases usually termed *infectious* are done, would be an imprudent measure. We must, however, endeavour to do justice to Mr. Blackadder: that he is a man of acute penetration, accurate discrimination, and nice judgment, is most clearly evident; and the work before us is an admirable specimen of what may be deduced from the application of such talents; but we consider the series of facts from which his conclusions are drawn are not sufficiently extensive to determine our opinion on the nature of the disease under every modification of circumstances, considering that the testimony of other judicious observers would lead to opposite conclusions.

Several species of ulcers are described by Dr. Jackson,* which he considers appear under such circumstances as if

* Sketch on Febrile Diseases, chap. iv. § 2.

they depended on some inexplicable modification in the action of the cause of fever—endemic or infectious. In particular places, and at particular seasons, they are so frequent as to be in a manner epidemic. We transcribe his account of the second species.—" The beginning of which is marked by the appearance of a pimple—hot and painful from the commencement, the pain sharp and stinging. The cuticle separates; the discharge is thin, sharp, and acrid. The circle of the pimple expands; the edges become red, and what is termed angry, with sensations of burning heat, sharp and pungent pain. The ulcerating process commences, and extends; the true skin is destroyed; the ulceration penetrates into the adjacent parts with more or less rapidity, affects the membrane of the bone, and, on many occasions, the bone itself. The discharge from these rapidly-spreading ulcers is sometimes thin and acrid, so as to excoriate, by its sharpness, sometimes copious, glairy, dusky-coloured, jelly-like, substance, more or less bloody; the surfaces underneath, and even the edges, are foul and fungous, or the adjacent parts break down rapidly into a disorganised mass." " This ulcerative form of fever is not common in the less healthy months of the year, where common fever most prevails, nor at the most unhealthy points of an unhealthy sea-coast. To this it may be added, that ulcer and formal fever do not ordinarily occur in the same place, much less in the same subject at the same time; but that, when the ulcer has healed, or when it has assumed a healing appearance, fever supervenes not unfrequently, and that it is mild and tractable when it does supervene, or violent and malignant in the last degree of malignancy, according to what had been the character of the preceding ulcer. It was the observation of this and other analogous facts, which induced me to rank the ulcerative form of disease, whether endemic or infectious, as I now do. The propriety of the arrangement may be disputed; I contend only for the accuracy of the fact as here stated."

We do not adduce this as hospital gangrene; but we consider that it may throw some light on the nature of that disease, which we have long considered, and have so expressed our opinion in various instances, as arising from the local effects of those causes, which, affecting persons without wounds, would produce what is usually termed typhous fever. If this notion be thought correct, it will explain the disputed point respecting the order in which the symptoms occur in gangrenous phagedena; as it will show that they may, like small-pox and some other diseases of this kind, take place in either way.

There is one important circumstance respecting hospital gangrene which we believe we have not mentioned, that of its recurring several times; this is noticed by all the practical writers on the subject. The sore will cease to spread; the slough become detached; and an appearance of healing take place: when, suddenly, without any evident cause, the peculiar morbid action will return, and the sore will again run through the process of inflammation and sloughing. It appears that, after a short time, the parts regain a susceptibility of the influence of the poison which may have been taken up from the sore, and then affects it through the medium of the general system. This is not consonant, as far as we are acquainted with the laws of those morbid poisons which affect the general system primarily, causing an acute attack of fever and secretion of a poisonous matter similar to that which produces the disease. The fever is considered by writers in general as acute or idiopathic, and of the typhoid character; but the description Mr. Blackadder gives of it, is that of fever symptomatic of local inflammation, or the irritation of the sore. This, with the repeated occurrence of the disease, is conformable to what we know of the law of those morbid poisons whose primary effects are local.

The author next treats on the method of cure, which resolves itself into two principal indications:—1st, to destroy the morbid action in the sore; 2dly, to regulate the re-action of the system. Our limits will not permit us to enter fully into the consideration of this part of the subject; and we have already mentioned the chief means of accomplishing it. There are a few circumstances, however, respecting it, on which we consider it necessary to make some observations. It has been stated that the external application of preparations of arsenic is not unattended with danger; Mr. Blackadder has never witnessed, but in one instance, any bad effects which could possibly be attributed to it; and this evidently arose from its having been applied in an improper manner. The French surgeons sometimes employ an arsenical paste; and instances are related of its employment having been followed by death, and of which it was apparently the cause. Mr. Blackadder considers that it is highly probable that the blame, in these instances, did not rest with the remedy, but the manner in which it was employed. It may be applied to a sore in such small quantities as to admit of its being absorbed into the system; but, by using it in sufficient quantity, quickly to destroy the organization of the part, such absorption may be prevented.

The sores should be kept perfectly clean by means of a solution of potash, and tow alone employed for this purpose, that every thing imbued with matter from them may be destroyed. Amputation has sometimes been had recourse to; but Mr. Blackadder states, that this was almost uniformly unsuccessful, the morbid action occurring in the stump. He then says,

"It may, therefore, be asked, if the disease can be removed by a topical application, acting merely as a cautery, why may not amputation answer the same purpose, seeing that the whole sore, and nidus of the disease, would thereby be effectually removed? And why should the disease again break out on the new surface of the stump, provided the patient be secured from any fresh source of contagion?"

To these questions, Mr. Blackadder replies, that the arsenical solution destroys the morbid action in the sore; yet, in many cases, the constitution must have become contaminated, by absorption of the poison; but we find that the natural powers of the system are such, on this as well as on other occasions, as to be able gradually to rid itself of this portion of morbific matter. Thus we find that, although the local morbid action be overcome by the arsenical solution, it will frequently recur again and again, requiring similar means for its removal; so that the arsenic may be said to arrest the local progress of the disease; and prevent a fresh production of morbid matter until the natural powers of the constitution are able to overcome the disease and effect a cure: or, as we should say, until the constitution is no longer susceptible of the irritation of the poison.

"The second indication of cure (says the author) will seldom require to be much attended to, if sufficient attention has been paid to the first symptoms of the disease: but, when the constitution demands attention, the re-action must be moderated or supported according to the plethoric or exhausted state of the patient."

We now arrive at the second part of the work, which contains an investigation into the history of gangrenous phagedena, as it is to be found in the writings of various ancient and modern authors. Mr. Blackadder here endeavours to prove, that the disease which forms the subject of this inquiry, far from being, as by many supposed, either new or overlooked, was not unknown; but, on the contrary, that its most appropriate treatment is distinctly pointed out by some of the oldest writers on surgery; and in this we consider Mr. Blackadder has succeeded. After mentioning the principles on which this inquiry was conducted, the author says,

"It is, nevertheless, in its present form, far from being what I could have wished, or what was intended, and is, indeed, only a small part of the materials that were collected with the hope of being able to lay something more worthy of perusal before my readers; but, owing to a state of health which little admits of that close or constant application, without which small progress or improvement can be made, either in literature or the sciences, I am forced either to place it, unfinished as it is, at the mercy of my readers, or what, to one who may have some professional zeal, and withal, not destitute of the ambition of being useful, is equally repugnant, to allow it to remain in its original obscurity. Those, who are accustomed to study their own minds, will at least not be surprised at the, perhaps unfortunate, alternative I have chosen."

We, who are not untried in those matters, well know the toil and difficulties he has undergone, and must bear testimony to our readers of the value of the fruits of his labours. We cannot, from the nature of the subject, give an account of the information it contains; and this we are less anxious to attempt, because we are confident that no one who has a due degree of zeal for the cultivation of surgery will neglect to make himself possessed of the work before us. We shall, however, take a cursory view of its contents, both because we feel a reluctance to dismiss such interesting matter unnoticed, and that the author has interspersed some remarks which we did not find in his practical history of gangrenous phagedena.

Mr. Blackadder commences with an effusion of admiration for men who now live the life of immortality; and a reprehension of folly and presumption, evidently dictated by a mind ardent with the love of nature and of truth.

He seems to consider the *Therioma* of Celsus, terminating in *Phagedæna*, as what he has described as phagedæna gangrænosa. The appearance of that ulcer without any evident cause, *(per se nascitur)* accompanied with a peculiarly disagreeable odour, stinging pains, extraordinary degree of surrounding inflammation, hæmorrhage, the rapid increase and extensive ravages it made, the fever which sometimes ensued, and the mode of cure by actual cautery in obstinate cases, appear to indicate its origin from and morbid poison; and the symptoms certainly bear a strong resemblance to those of hospital gangrene. Roland and Belloste, in particular, appear to have witnessed the disease; the remedies they employed were composed of lime, colcothar of vitriol, and arsenic. Some valuable observations and extracts from the works of Rogerus, Brunus, Lanfrancus, Guido, and Ferrus, ensue. Ambrose Parey mentions a state of wounds after injuries, both from gun-shot and swords, which has some of the characters of hospital gangrene; but he is not very particular in his description, considering it to arise from a corrupted state of the atmosphere. His remedy was the unguentum Ægyptiacum. We pass over Wiseman and

Horstius to La Motte, who distinctly describes it as occurring at the Hôtel Dieu. His remedies were Ægyptiacum, storax, and other warm and spirituous applications. In the posthumous works of M. Poutean are two memoirs on this disease; he considered the disease as contagious, and the consequences he witnessed from it such as to make him ask, "Have hospitals been more beneficial or pernicious to human nature." He employed the actual cautery; or, if the surgeon or patient were so timid as to be terrified at the use of this remedy, boiling oil, with the addition of some acrid substances might be substituted for it. He then notices the paper written by Dr. Gillespie on this disease. The observations of Dr. Adams on *Sloughing Phagedena,* in his work on Morbid Poisons, are next referred to. On these, Mr. Blackadder makes no remarks, considering that every one is familiar with the work; and because (in the words of the author) "it is next to impossible to do justice to him, without making much larger extracts than is, on the present occasion, admissible; more particularly as my attention is principally directed to the practical observations of such as have themselves been actors on the stage." Here we consider Mr. Blackadder has fallen into an error; it is evident to us that Dr. Adams did not consider the disease he termed sloughing phagedena as hospital gangrene; and, therefore, his observations are not at all applicable to this question.

Some remarks ensue on the memoir of Citizens Moreau and Burdin. The account Dr. Rollo published of this disease, at the end of his Treatise on Diabetes, as it occurred at the Military Hospital at Woolwich, with which we suppose our readers are well acquainted, is next considered. The observations of this gentleman on the nature and progress of the disease, and his principles for the method of cure, very nearly accord with those of Mr. Blackadder. We can merely notice the names of the authors who have subsequently written on the subject, which are—Mr. Ballard, Dr. Harness, (whose observations were communicated to the 4th volume of our Journal,) Mr. Edwards, Mr. John Bell, Dr. Trotter, Mr. Caird, Mr. Brown, Mr. M'Dowal, Mr. Arthur, Mr. Little, M. Larrey, M. Delpech, and Dr. Thomson.

In the course of his remarks on the work of M. Delpech, the author says,

"I do not wish it to be understood that I believe it impossible for this contagion to be, in any given circumstance, conveyed to a sore through the medium of the atmosphere; but there cannot be a doubt, that by far the greater bulk of the evidence we are possessed of goes to support an opposite view of the subject; and, therefore, I contend, that the facts which have hitherto been brought forward in support of this mode of propagation, being in no instance decisive, can at most be only considered as exceptions to a general rule. I have frequently had occasion to remark, on removing fomentations or poultices, which had been applied to sores affected with this disease, and even when the temperature of the atmosphere was upwards of 60° Fahrenheit, that a dense white vapour arose, and was in some instances visible upwards of six inches above the sore. It seems, therefore, possible, that a small or crowded apartment may, through the ignorance or negligence of the medical attendant, be made to resemble a vapour-bath, by the effluvia from the sores being allowed to accumulate so as to be almost visible. In such a case it is not improbable, that the disease would be propagated through the medium of the atmosphere. This, however, I would consider equivalent to inoculation or immediate contact."

It is obvious that those, whose observations have led them to consider the general system as primarily affected, could with equal propriety apply this mode of reasoning to favour their opinions respecting the manner in which the disease may be disseminated. We must, however, remark that the character of the sore appears more to resemble that arising from local contact of a morbid poison, than from specific action consequent on general disease of the system.

Our limits will not permit us to dwell any longer on the consideration of this work; but we cannot pass over the remarks of Mr. Blackadder on the observations of Dr. Thompson on hospital gangrene, without declaring our approbation of them; the strength and dignity of manner, with which they are expressed, indicate great delicacy of feeling, and a well-cultivated mind.

Energy of style, polished wit, and well-directed raillery, are highly grateful to the critic, and soothe his toils in pointing out the paths which lead to the dwelling of science; and we have found them in the work, the review of which we have just completed. But these are not the traits with which the merit of it should be depicted: it has a more noble and important character,—that of eminently contributing to improve the art of surgery.

---

*Practical Observations on the Action of Morbid Sympathies, as included in the Pathology of certain Diseases: in a Series of Letters to his Son, on his leaving the University of Edinburgh, in the Year* 1809. By ANDREW WILSON, M. D. Kelso. pp. 406. Edinburgh.

MEDICINE, more fertile perhaps in theories, and more mutable than other sciences, because the phenomena about which it is engaged are more complex, and dependant on actions that lie concealed from the cognizance of sense, is daily strengthened by the acceded stores of experience. Theory, as it precedes the ample accumulation of facts in any science, is necessarily imperfect; there being more links in the chain for conjecture to supply. Erroneous in its outset, it progressively corrects itself by the gradual extension of established data. The more correct views of enlarged experience sometimes raise a smile at the fanciful hypotheses of earlier philosophy, whilst they glean from her recorded data materials for the establishment of a depurated system. A fact nicely observed and accurately pourtrayed, or experience faithfully registered, though blended with some untenable hypothesis for their explication, is still a tribute of value to the stores of science. The philosopher of future ages is a debtor to the faithful historian of nature.

The work before us, as is stated in the Preface, originated in a series of letters addressed, some few years back, by the author to his son, who was then closing his medical pupillage. The object of this correspondence was to fix the attention of a young practitioner on certain important phenomena, which, if truly methodised, and associated in their natural relations, might afford him a clue to trace the mazes of the therapeutic labyrinth.

Diagnostic pathology is the subject which Dr. Wilson is solicitous to impress forcibly on the close attention of his correspondent; as on the accuracy of his views of the dependencies of morbid actions rest the success or failure of a practitioner in his endeavours to remove disease. His own particular opinions on certain points in pathology, as having grown out of long observation and reflection, the author considers to be fitted to assist the judgment, and facilitate the practice, of his son in his future professional career. At all events, these opinions, when brought into contact with those of other men, may serve as reciprocal correctives.

The indefinite language and vague theories which deform medical writings have arisen, as he says, from the obscurity and intricacy of the subjects, and the paucity of established data on which to found legitimate deductions. Yet there are not wanting certain fixed principles, inherent in the living body, which are always present, either in real action, or ready to be called into action.

"This is very observable in the well-known laws of that general sympathy inherent in the whole nervous system; as is demonstrated by the fact, that an injury offered to any part of that system can communicate a morbid sensation either to the whole or to some individual organ, in which the nervous influence is at the time supporting a state of health."

The ascertained laws of these sympathies, the author thinks, may be regarded as principles on which reasoning may be safely grounded. He adduces, in support, the constancy of certain concomitant sympathies, in cases of known sources of irritation; whence the existence of the primary offending cause may be inferred, from the presence of the sympathetic action.

"Placed by the Creator as a medium of connection betwixt the living principle and the mere mechanical part of the human body, the nervous fabric, consisting of the brain and its appendages, constitutes a most important field of attention," &c. &c.

This implies that life is a something superadded to organisation, which holds a more immediate communion with that part of our system which we denominate brain and nerves. Without entering into the consideration of this not universally-admitted doctrine, we merely quote the passage "*en passant*," as giving a view which will probably influence some of the subsequent reasonings of the author.

"The existence of the law of general sympathy, which is inherent in the animal economy, has long been known and taught in the physiological schools; and many also are the specific sympathies of one individual organ with another, which have been remarked to exist during a state of health. But it is the exercise of this law, in its extensive and various forms, under a diseased state of the system, to which I propose chiefly to lead your attention, as affording a satisfactory explanation of many morbid appearances; the nature of which, I am led to think, cannot be demonstrated so clearly, nor so intelligibly, on any other ground."

In this passage, Dr. Wilson prescribes the scope and object of his work, which we therefore give in his own words. And we are of opinion that no subject possesses a larger claim on the attention of the medical practitioner.

An interruption in the healthy function of any organ necessarily induces some disorder in the actions of that part of the system to which this function is necessary. The disorder is sometimes so apparent as to lead us directly to the source of disease; but, in many instances, both the effect and cause are so obscure as to elude our observation. The existence of disease being manifest, we are often obliged to guess at its seat and nature by remote effects. Here it is that we must call to our assistance the operation of the general laws of nervous sympathies and, though they have been little used in pathological reasoning, they will prove of important practical utility. In these general laws, the author again asserts, we shall find principles so fixed, that we may reason on them almost with the certainty of axioms, and act upon them with confidence.

"The brain, and its appendages of nerves, being that medium through which the living principle acts upon the otherwise inert organic structure, as also the medium through which the mind is made conscious of injurious applications offered to any part of the machine, it may properly be said that every part of the human body is capable of sympathising with the rest; because we see that no part of it can admit of having the sense of feeling excited to the height of pain, without the whole frame also suffering an uneasy sensation."

This passage, in accounting for a fact by an hypothesis, shows how readily, and even unconsciously, we may arrive at false conclusions, through a legitimate course of induction, by reasoning on an assumption as on an incontrovertible principle. The nervous system, we have strong evidence to admit, is, in the animal economy, the medium of sensation, the medium of organic action, of motion, of passion, of consciousness, of intelligence. This we have reason on good data to believe; but how it is so we are still unprepared to explain. We have surely no warrant for saying, that it is because the brain and nerves are instruments through which inert organic structure is acted on by an independent living principle; for the existence of such an abstract principle is purely hypothetical. It is a gratuitous assumption to surmount a difficulty. The brain and nerves are organised, as are all other parts of the machine, and composed of matter as inert,—if by inertness we understand, in matter, an incapacity for self-motion. But all matter is mobile and moving, for on the motion of matter rest the phenomena of the universe!

The brain and nerves, independently of their office in the system, have organic action, as have other parts. Are they not produced, nourished, absorbed, replaced, by the actions of other systems in the frame? Does not their very function depend on the due maintenance of those actions in other parts of the system which they are supposed to govern? Suspend those agencies by which foreign matter is brought to exercise its appropriate influence on the material mass of a living being, and what becomes of nervous power, and of the independent living principle? Is it to account for mechanical motions in vital beings, that we are to have recourse to the influence of an imaginary indefinable existence? Are the motions of attraction and repulsion in inorganic matter the effects of a vital principle? Is the shock transmitted through a series of contiguous bodies, by an electric explosion, to be accounted for by the supposition of a vital principle? The influence of powerful agents is transmitted through certain media, in a manner not exposed to our senses, in every display of the phenomena of electricity, of galvanism, and of magnetism. The pile of Volta exhibits to us the production, the accumulation, the motion, of an intense excitant, from the chemical interchange of elementary constituents. What is there then in the material actions of a vital being which differs from the powers and agencies of inorganic matter? Life is a connected series of reciprocally-productive and mutually-dependant actions, chemical and mechanical, which support, through a limited period, the functions and fabric of an individual organization by the continued change and excretion of internal, and by the assimilation to itself of foreign matter. These actions are, in the most simple organized structure, of exceeding complexity; but they are actions which we see displayed, though disunited, in the perpetual interchange of principles in inanimate substances.

Though we do not admit the whole of Dr. W.'s physiology of the nervous system, we fully agree with him, that in this part of the animal economy must be considered to reside the capacity for sensation and for sympathies. The brain and nerves form as it were an integrant system within the animal structure, pervading minutely every portion of it, and referring by its universality the actions of every part to a common focus; it balances, doubtless, the healthy actions of the whole system: perhaps, it conveys the excitement of one chemical mutation to rouse action in another part. It receives, it conducts, it accumulates, it diffuses the principle of excitation. It is probable that, by the mediation of nervous structure, organic mechanical motion, whereby propulsion of fluids through the tubular apparatus is effected, is derived from the influence of chemical agencies. But experiments are still wanting to establish an entire theory of the agencies of this important portion of the animal machine.

That the whole system is influenced by sympathies in a healthy state, appears in the general effects following the momentary condition of a part. If the stomach be duly stimulated by food, or its accustomed pabulum be withheld, does not energy or languor pervade the whole? Muscular exertion or inaction, the agreeable or offensive excitement of an organ of sense, will raise or depress the actions of the entire system. But, besides these sympathies, which are not so much the object of consideration to the pathologist, there appears to be a consentaneous action in parts where some deviation from a healthy state has locally occurred; thus, deranged function or morbid action in one part of the system is commonly followed or accompanied by disturbance in some other. It is to the contemplation of these morbid sympathies, which open a field of much interest, that the author expressly calls the reader's attention in the present volume. After alluding to the general sympathetic connexion of the animal frame, he says,

"But there are likewise parts or organs which are more intimately connected with and dependant on each other, than upon the rest of the body, constituting a specific or peculiar sympathetic affection."

Of these he gives ordinary instances; as of the stomach and head, the uterus and stomach; and ascribes them to a general law of the sentient nervous system He then proceeds to show, that the sympathetic affection is sometimes attended with pain, sometimes not; sometimes only an increase or diminution of the healthy action of the sympathising organ results from the original irritation.

In accounting for pain as a concomitant of morbid sympathy, the author is lead to ascribe it to spasm affecting muscular structure or even fibrous membrane. Where a nerve, a plexus, or a ganglion, is the seat of pain, it is necessarily very great.

"But, as the medullary part of the nervous structure is possessed of no contractility, therefore, if it is supposable that a sympathetic affection is ever exclusively seated on that part of the chord, it is to be presumed that the attending pain may arise from a morbid excitement of its natural sentient principle. But, although the nervous medulla has in itself no contractility, yet its membranous sheaths are possessed of muscular fibrillaæ, and blood vessels, like other membranes; and, therefore, may be affected with sympathetic spasms, so as to influence the medullary part with painful sensations. There is reason to believe that this in fact occurs."

Cerebral medullary substance is truly almost the only part of our devoted system-tortured frame that has escaped the ubiquitous gripe of spasm; and even this, as it slides through the reluctant fingers of our author, hardly escapes a malicious pinch. The investing sheaths of nerves, we were taught, were continuous productions from the meninges of the brain; and, if so, we do not comprehend how they can be the seat of spasm. They are supplied with blood vessels, it is true, for their nutrition; yet, admitting for a moment that the slender tunics of these minute *arteriolæ arteriarum* could come within the grasp of this petty tetanus, could that produce compression on the medullary contents of the sheath? But there is something in this doctrine of capillary cramp too refined for slow apprehensions and ordinary optics.

Pain may be caused by irritation of medullary substance, and probably by mere mechanical compression, for which we are not to look alone to spasmodic stricture. An over distension of vessels, or forcible extension of yielding structures, will necessarily compress interposed nerve.

Voluntary muscular contractions are unattended by pain: spasmodic contractions are not. But spasm is only an inordinate degree of contraction, excited by irritation, remote or immediate. Whenever, therefore, muscular fibres exist, morbid spasm may also exist:

"Consequently, not only the fibres of large muscles, but the smallest fibrous threads of all the membranous parts, are also susceptible of spasm, from irritation applied either directly or indirectly from morbid sympathy; and hence spasm, when fixed in the fibres of a membrane by sympathetic affinity, may be productive of painful sensation, as naturally as when seated in the fibres of a large muscle."

Because spasm affects muscular fibre, it may affect membranous fibre; and, as spasm gives pain in muscles, it may give pain in membranes.

We cannot conceive how any thing at all analogous to the inordinate, forcible, involuntary, but not unnatural, contraction of a muscle, which we designate by the term Spasm, can by any possibility have place in a purely membranous structure. Are the dura mater, the pleura, and the peritoneum, motive organs? are they expansive and contractile by stimulus? were they ever thought, under any circumstances, to draw themselves into rugæ, or to compress an enveloped viscus? Surely the term spasm is here vaguely applied to account for an affection not understood.

Assuming, then, not merely the possibility, but the actual existence, of membranous spasm, the author proceeds to remark, that, contrary to the ordinary consequences of violent spasmodic action in large muscles, in membranes it is speedily followed by local inflammation.

"Yet, when the muscular texture of a membrane becomes the seat of a sympathetic spasm, local inflammation of that membrane is speedily induced; the coats of its capillary vessels being probably also soon included in the sympathetic influence."

Is there no way of accounting for inflammation in a membrane, but from sympathetic spasm? and the coats of its capillary vessels!!!

"Instances of this frequently occur in spasm of the intestines, this spasm, by the way, does not occur in the simply membranous structure of these organs, and are often exhibited to the eyesight in periodical head-ach, more especially if the periodical sympathetic pain extends to the membranes of the eye. I have, in that case, observed the adnata become regularly inflamed during the continuance of the fit, disappearing gradually in the time of the intermission, and returning again with the returning paroxysm; at other times, on such occasions, the inflammation is more permanent, and establishes a most distressing form of ophthalmia."

To our actual inspection, indeed, is often presented, in diseases of the exposed membranes of the eye, the visible phenomena of inflammation. Does not this present a fair field, too, in which to witness its precursor, "sympathetic membranous spasm?"

Following this general reasoning, the author proceeds—

"Here, then, we have a certain number of pathological facts, which must be admitted as data to be reasoned from, because their existence is demonstrably true, and, I believe, are as certainly so as any axiom in geometry.—1st. Irritation, applied to certain parts of the body, can excite painful sensations and morbid action in a distant organ, by the operation of the law of sympathy inherent in the nervous system: this frequently occurring whilst the mind remains unconscious of any uneasy feelings at the place where the irritation is applied. 2nd. Morbid sympathies are sometimes attended with pain, and sometimes not. In the latter case, the morbid effect consists either merely of an augmented action of the natural powers of the sympathising organ, or else, as happens at other times, the natural healthy powers of the organ are thereby impaired. But, when an action of morbid sympathy rises to the height of exciting involuntary spasm in a muscle, in a portion of a muscle, or in the fibrous texture of a membrane, it is uniformly attended with more or less of painful sensations in the sympathising organ, and there occasionally in a severe degree. 3d. When a membrane is the seat of sympathetic spasm, the circulation in that membrane is frequently influenced in such a manner as speedily to produce inflammation, more especially in those of a firm texture."

The forms and appearances of morbid sympathies vary.

"The original irritating cause will at one time produce a single peculiar sympathy only; and at another time will give a number of sympathising affections, occupying several organs of the body at the same time, and all of these appearing to flow from the same source; as, for instance, the many forms of uneasy sensation which can be traced as resulting, at one and the same time, from vitiated fluids contained in the stomach.

"Upon other occasions, the effects of the primary cause are progressive, and the first sympathetic affection will produce a second depending upon it; and this again will give a third, in like manner; and so on in a series of morbid sympathies flowing in succession from each other, and exhibiting morbid appearances peculiar to the organs included in the climax. This is demonstrated when irritation is excited in the kidneys or bladder by a stone or gravel; or in the uterus in some forms of hysteria, and in the case of pregnant women; which organs have a peculiar sympathetic affinity with the stomach and intestines. But the stomach again readily communicates its morbid influence to the head; and there, if the morbid sympathy shall be seated in the cranium, there is no organ nor function which may not be included in the circle: the whole phenomena, at the same time, depending on the primary irritation subsisting in the urinary organs or uterus. And further, you must observe, that the painful sympathetic spasm very often shifts its seat; which appears to be the effect of some unperceived natural cause, or, as sometimes happens, from the agency of a medical application made to the parts. But, the original cause of irritation still remaining in action, the effect of that cause merely changes its place to some other organ, which may stand next in rank of affinity to the primary cause; and soon to a third, perhaps, or even return to the first, as circumstances attending the original cause may determine: but this will appear more forcibly to you in considering the sympathetic phenomena of certain diseases afterwards. With regard to the vinculum connecting the sympathising part with that where the irritation is applied we remain totally in the dark: all that we have in our power, on this point, is to observe, and by careful observation to remark, demonstrable facts; and these, by candid induction, ought to lead to correct practice. Neither is it known whether the communication of sympathies be direct, being established by the various subdivisions of the nervous chords; or if these communications are circuitous, always passing first to the brain, and so, by the medium of the sensorium commune, exhibiting these phenomena which appear in the sympathising organs."

*(To be continued.)*

